# T139. ELECTRORETINOGRAM ABNOMALIES IN SCHIZOPHRENIA PATIENTS WITH VISUAL HALLUCINATIONS

**DOI:** 10.1093/schbul/sby016.415

**Published:** 2018-04-01

**Authors:** Vincent Laprevote, Florent Bernardin, Thomas Schwitzer, Raymund Schwan

**Affiliations:** Centre Psychothérapique de Nancy

## Abstract

**Background:**

Retinal dysfunctions have been integrated in cognitive models of visual hallucinations in several pathologies such as Parkinsonian syndromes or eye diseases. Besides, structural abnormalities of the retinal ganglion cells are documented in schizophrenia and have been associated to visual hallucinations (VH) in neurological disorders. We aim to study functional abnormalities of retinal ganglion cells in schizophrenia patients with VH.

**Methods:**

We measured the activity of retinal ganglion cells using electroretinogram according to ISCEV criteria. We compared the amplitude and implicit time of the P50 and the N95 waves of the pattern electroretinogram in schizophrenia patients with VH (VH group, n = 7), Auditory Hallucinations or no hallucination (AH/NH group, n = 8) and controls (n = 30).

**Results:**

Preliminary findings show a significant increase of the N95 implicit time in the HV group compared with controls (p = .05). No difference was found between the HV and HA/NH groups but a gradient appeared to emerge between the 3 groups.

**Discussion:**

Functional impairment of the retinal ganglion cells appears to be more pronounced in schizophrenia patients with HV. The increase of N95 implicit time may be interpreted as a dysfunction of retinal ganglion celles rather than a cell loss. These preliminary results need to be confirmed with a larger sample.

